# *In vivo* Confocal Microscopic Evaluation of Previously Neglected Oval Cells in Corneal Nerve Vortex: An Inflammatory Indicator of Dry Eye Disease

**DOI:** 10.3389/fmed.2022.906219

**Published:** 2022-06-03

**Authors:** Dalan Jing, Xiaodan Jiang, Yilin Chou, Shanshan Wei, Ran Hao, Jie Su, Xuemin Li

**Affiliations:** ^1^Department of Ophthalmology, Peking University Third Hospital, Beijing, China; ^2^Beijing Key Laboratory of Restoration of Damaged Ocular Nerve, Peking University Third Hospital, Beijing, China; ^3^Department of Ophthalmology, BenQ Medical Center, The Affiliated BenQ Hospital of Nanjing Medical University, Beijing, China; ^4^Beijing Ophthalmology and Visual Sciences Key Laboratory, Beijing Tongren Eye Center, Beijing Tongren Hospital, Capital Medical University, Beijing, China

**Keywords:** dry eye disease (DED), corneal vortex, Langerhans cells, *in vivo* confocal microscopy (IVCM), artificial intelligence (AI)

## Abstract

This study aimed to investigate the association of between previously neglected oval cells located in the corneal vortex and dry eye disease (DED). This was an observational, prospective study involving 168 patients with different degrees of DED. *In vivo* confocal microscopy was used to observe the corneal subbasal nerves and Langerhans cells (LCs) in the corneal vortex and periphery. Bright and oval cells were also observed in the corneal vortex. An artificial intelligence technique was used to generate subbasal nerve fiber parameters. The patients were divided into the three groups based on the presence of inflammatory cells. Group 2 patients showed a significant increase in the corneal peripheral nerve maximum length and average corneal peripheral nerve density. Patients in group 3 had more LCs than other patients. A bright and oval cell was identified in the corneal vortex, which might be a type of immature LC related to the disease severity of DED.

## Introduction

Dry eye disease (DED) is a worldwide epidemic, with a prevalence of 5–35% in the different age groups (1). Dry eye is multifactorial in origin and is characterized by loss of tear film homeostasis. The accompanying ocular symptoms vary. Tear film instability and hyperosmolarity, ocular surface inflammation and damage, and abnormal neurosensory function are the main causes of DED (2). Traditionally, investigation has required the ocular surface disease index (OSDI), tear breakup time (TBUT), corneal fluorescein staining, Schirmer’s test, and tear osmolality. These series of investigations are usually used to assess the severity. However, direct information on the inflammatory activity provided by these methods is very poor. Inflammation is a recognized component of the pathophysiological mechanism of DED (2) and has been proposed as a stable indicator of DED severity (3). *In vivo* confocal microscopy (IVCM) is a new non-invasive technique for displaying the microscopic morphology of the cornea at the cellular level, such as the corneal epithelium, corneal nerve, and inflammatory cells (4), while achieving satisfactory resolution and allowing timely repeatable inspection (5). The subbasal corneal nerve plexus is the most densely distributed corneal nerves, located between the basal layer and Bowman’s membrane, and is the most studied structure (6). The effects of dry eye on the corneal subbasal nerve plexus have been controversial in various studies (7–9). Several explanations may account for these differences, such as the severity of illness, classification of disease, or examination of the corneal location (10). Previous studies have usually measured the central cornea using IVCM; however, due to less observed range and positioning function, the results of each measurement were obtained in different positions, with increased variability, poor reproducibility, and decreased comparability. Therefore, an effective and reasonable location for examination is required. The corneal vortex is a unique structure recognized in recent years and is commonly found in people (11). Studies have shown a radiating pattern of nerve fiber bundles converging toward an area approximately 1–2 mm inferior or nasal to the corneal apex in a whorl-like pattern (12). Additionally, the corneal vortex is easy to recognize and has a relatively fixed site compared to the corneal center (13). Therefore, the corneal nerves were divided into two parts: the corneal vortex and the corneal periphery. Previous studies have shown that dry eye is a chronic CD4 (+) T cell-mediated autoimmune inflammatory disease of the ocular surface. As the first-line sentinel, Langerhans cells (LCs) play a role in ocular surface inflammation (14). Lin et al. (15) found that the central corneal LC density may indicate dry eye severity. Recently, a type of bright, oval cell was found at the vortex of the subepithelial nerve plexus distributed in the corneal vortex in IVCM images. Previous studies have shown this type of cell, but no further studies have been reported (11, 16). They were considered as immature LCs and the severity of DED was associated with them. To test this hypothesis, 168 patients were recruited and the effect of these cells on DED was analyzed.

## Materials and Methods

### Subjects

The Ethics Committee of Peking University Third Hospital approved this study. The study was conducted in accordance with the principles of the Declaration of Helsinki (#M2019236). Written informed consent was obtained from all the patients before the inspection.

This prospective study included 168 patients (109 women and 59 men; mean age, 69.00 ± 9.10 years; age range, 60–89 years) with different degrees of DED diagnosed based on the 2017 Report of the Tear Film and Ocular Surface Society International Dry Eye Workshop II (TFOS DEWS II) (17). Patients with symptoms of dryness, foreign body sensation, burning, fatigue, blurred vision, and TBUT < 10 s were recruited. The exclusion criteria were: any ocular surface and corneal abnormalities; history of contact lens wear within 1 month; recent eye surgery; nasolacrimal duct obstruction; glaucoma; ocular fundus diseases; diabetes; and systemic immunologic disease, including systemic lupus erythematosus and rheumatoid arthritis. The right eye was majorly enrolled; the other eye was also included, if the right eye did not meet the inclusion criteria.

### Dry Eye Examinations

We conducted clinical assessments of the enrolled patients in the following order: a collection of demographic information, the Ocular Surface Disease Index (OSDI) scores, oculus, and IVCM. The OSDI questionnaire was used to describe subjective eye-related discomfort (18). The central tear meniscus height (TMH) of the lower eyelid, TBUT, and the meibomian gland (MG) morphology of both the lower and upper eyelids were recorded using Keratograph 5M (OCULUS, Wetzlar, Germany). The same technologist performed the examination and the TMH and MG morphology outcomes were further analyzed by the same ophthalmologist.

### *In vivo* Confocal Microscopy

Images obtained using IVCM (HRT II RCM Heidelberg Engineering Incorporation, Heidelberg, Germany, Rostock Cornea Module) had a definition of 384 pixels × 384 pixels over an area of 400 μm × 400 μm, with a lateral spatial resolution of 0.5 μm and a depth resolution of 1–2 μm (19). IVCM was performed on each eye in two different areas: the corneal vortex and the superior peripheral cornea. The periphery was defined as the one-sixth outside part of the cornea (20). Approximately, 30 images were captured from the corneal epithelium to the endothelium and good-quality images were selected for analysis.

### Corneal Image Analysis

Five high-quality images with no overlap in the corneal vortex and periphery were selected for analysis of the corneal nerve parameters using corneal nerve segmentation network (CNS-Net). Images should reveal at least one visible corneal nerve and those with strong artifacts were excluded (21). A mathematical model can repeatedly perform nerve tracing and evaluation, thus achieving relatively stable and consistent results (21). The corneal nerve parameters obtained are maximum length of the corneal vortical nerve, average density of the corneal vortical nerve, maximum length of the corneal peripheral nerve, and the average density of the corneal peripheral nerve. Previously, bright irregular particles or undefined dendritic structures, which appeared scattered among nerve fibers, were regarded as the LCs (22). However, quite apart from this, the bright, oval cells were included as LCs in this study. The LCs of the selected images were counted and their average was calculated to determine the LC number. Moreover, five other selected images with more LCs were counted and their average was calculated to determine the average LC number of the total corneal nerve.

Patients were divided into three groups: group 1 with no LCs; group 2 had both the LCs and bright, oval cells; and group 3 had LCs but no bright, oval cells.

### Statistical Analysis

All the statistical analyses were performed using SPSS version 23.0 software. We verified the normality of the data distribution using the Kolmogorov–Smirnov test. Descriptive parameters are expressed as the number of patients (%) or mean ± SD or median with interquartile range, depending on the distribution pattern.

Continuous variables were compared with ANOVA or the Kruskal–Wallis tests. Continuous data among the groups were compared using independent *t*-tests or the Mann–Whitney non-parametric *U*-tests. Categorical variables were compared using the chi-squared test. Differences in LC numbers, corneal nerve average density, and corneal nerve maximum length between the vortex and the cornea’s periphery were analyzed using the paired Student’s *t*-test. Correlation analyses between clinical variables were performed using Spearman’s index of linear correlation. *P*-value < 0.05 was considered significant for all the comparisons.

## Results

### Patients’ Demographics and Ocular Characteristics

A total of 168 subjects were enrolled in this study, with 30 patients in group 1, 78 patients in group 2, and 60 patients in group 3. The age and sex of each group were matched (*P* > 0.05). No differences in the TMH, TBUT, and MG scores were found among the groups (*P* = 0.31, *P* = 0.66, and *P* = 0.22, respectively). The mean OSDI score of all the patients with DED in this study was 34.87 ± 21.80. The demographic and ocular characteristics of the three groups are shown in Table 1.

**TABLE 1:** Demographics and ocular characteristics of the subjects in three groups.

Item	Group 1 (*n* = 30)	Group 2 (*n* = 78)	Group 3 (*n* = 60)	*P*	Total
Sex					
Male (*n*)	8	23	28		59
Female (*n*)	22	55	32	0.06	109
Age (years)	66.90 ± 8.53	69.30 ± 9.00	69.67 ± 9.45	0.39	69.00 ± 9.10
OSDI score	33.80 ± 27.71	35.81 ± 22.09	33.69 ± 19.79	0.75	34.87 ± 21.80
TMH (mm)	0.18 ± 0.02	0.20 ± 0.07	0.18 ± 0.07	0.31	0.19 ± 0.06
TBUT (s)	4.59 ± 1.53	4.60 ± 2.03	4.69 ± 2.41	0.66	4.63 ± 2.09
MG score	3 (2.3)	3 (2.3)	3 (2.4)	0.22	3 (2.3)

*OSDI, Ocular Surface Disease Index; TMH, tear meniscus height; TBUT, tear film break-up time.*

*P-values of less than 0.05 were considered statistically significant.*

### *In vivo* Confocal Microscopy Examination Results

The corneal peripheral nerve maximum length and average density were significantly different among the three groups (*P* = 0.041, *P* = 0.036, respectively). The results of the maximum length of the corneal vortical nerve and the average density of the corneal vortical nerve, the maximum length of the corneal peripheral nerve and the average density of the corneal peripheral nerve, the average LC number of the corneal vortex, the average LC number of the corneal periphery, and the average LC number of total corneas are shown in Table 2.

**TABLE 2:** Corneal sub-basal nerve parameters of the subjects.

	Group 1 (*n* = 30)	Group 2 (*n* = 78)	Group 3 (*n* = 60)	*P*
Corneal vortical nerve maximum length (mm)	2.71 ± 0.60	2.70 ± 0.73	2.47 ± 0.71	0.158
Corneal vortical nerve average density (mm/mm^2^)	15.82 ± 3.47	15.89 ± 4.49	14.53 ± 4.07	0.146
Corneal peripheral nerve maximum length (mm)	3.20 ± 0.52	3.27 ± 0.62	3.02 ± 0.62	0.041*
Corneal peripheral nerve average density (mm/mm^2^)	16.73 ± 2.89	17.54 ± 3.62	15.90 ± 3.65	0.036*
Average LC number of the corneal vortex (number)	0	1.90 ± 0.92	1.93 ± 0.90	0*
Average LC number of the corneal periphery (number)	0.90 ± 1.12	1.68 ± 0.93	2.22 ± 0.88	0*
Average LC number of total corneal (number)	5.56 ± 4.56	11.29 ± 10.62	21.12 ± 12.84	0*

*LC, Langerhans cell.*

P-values of less than 0.05 were considered statistically significant. *Significant correlation (P < 0.05).

### Comparison of Vortical and Peripheral Corneal Parameters

The average density and maximum length of the corneal nerve in the peripheral area were significantly greater than those in the vortical area (*P* < 0.01). However, there was no difference in LC number between the two areas (*P* = 0.12). Additional details are given in Table 3.

**TABLE 3:** Comparison of vortical and peripheral corneal parameters.

	Peripheral area	Vortical area	*P*
Corneal nerve maximum length (mm)	3.17 ± 0.61	2.62 ± 0.71	0*
Corneal nerve average density (mm/mm^2^)	16.86 ± 3.53	15.39 ± 4.21	0*
Average LC number (number)	1.73 ± 1.05	1.66 ± 1.12	0.12

LC, Langerhans cell. P-values of less than 0.05 were considered statistically significant. *Significant correlation (P < 0.05).

### Correlation Analysis of Subbasal Nerve Parameters and Ocular Features of Each Group

The correlation coefficients (CCs) of nerve parameters and ocular features in patients with DED are given in Table 4. In group 1, the OSDI score and poor vision were positively correlated with the average LC number of the corneal periphery (CC = 0.74, 0.82, respectively, all *P* < 0.05). Furthermore, poor vision was positively correlated with the average LC number of the total corneas (CC = 0.77, *P* < 0.05). Blurred vision and TMH were positively correlated with the maximum length (CC = 0.88, 0.88, respectively, all *P* < 0.05) and average density (CC = 0.54, 0.58, respectively, all *P* < 0.05) of the corneal vortical nerve. However, TBUT was negatively correlated with the average LC number of the corneal vortex and total cornea in group 2 (CC = 0.33, 0.27, respectively, *P* < 0.05). In group 3, the OSDI score and foreign body sensation were significantly correlated with the average LC number of total corneas, with a CC of 0.41 (*P* < 0.05) and 0.54 (*P* < 0.05), respectively. Moreover, foreign body sensation was positively correlated with the average LC number at the corneal periphery (CC = 0.42, *P* < 0.05).

**TABLE 4:** Correlation of corneal nerve parameters and ocular features.

		OSDI score	Sensitivity to light	Foreign body sensation	Painful eye	Blurred vision	Poor vision	TMH	TBUT	MG score
Group 1	Corneal vortical nerve maximum length (mm)	NS	NS	NS	NS	0.88*	NS	0.54*	NS	NS
	Corneal vortical nerve average density (mm/mm^2^)	NS	NS	NS	NS	0.88*	NS	0.58*	NS	NS
	Corneal peripheral nerve maximum length (mm)	NS	NS	NS	NS	NS	NS	NS	NS	NS
	Corneal peripheral nerve average density (mm/mm^2^)	NS	NS	NS	NS	NS	NS	NS	NS	NS
	Average LC number of the corneal vortex (number)	NS	NS	NS	NS	NS	NS	NS	NS	NS
	Average LC number of the corneal periphery (number)	0.74*	NS	NS	NS	NS	0.82*	NS	NS	NS
	Average LC number of total cornea (number)	NS	NS	NS	NS	NS	0.77*	NS	NS	NS
Group 2	Corneal vortical nerve maximum length (mm)	NS	NS	NS	NS	NS	NS	NS	NS	NS
	Corneal vortical nerve average density (mm/mm^2^)	NS	NS	NS	NS	NS	NS	NS	NS	NS
	Corneal peripheral nerve maximum length (mm)	NS	NS	NS	NS	NS	NS	NS	NS	NS
	Corneal peripheral nerve average density (mm/mm^2^)	NS	NS	NS	NS	NS	NS	NS	NS	NS
	Average LC number of the corneal vortex (number)	NS	NS	NS	NS	NS	NS	NS	−0.33*	NS
	Average LC number of the corneal periphery (number)	NS	NS	NS	NS	NS	NS	NS	NS	NS
	Average LC number of total cornea (number)	NS	NS	NS	NS	NS	NS	NS	−0.27*	NS
Group 3	Corneal vortical nerve maximum length (mm)	NS	NS	NS	NS	NS	NS	NS	NS	NS
	Corneal vortical nerve average density (mm/mm^2^)	NS	NS	NS	NS	NS	NS	NS	NS	NS
	Corneal peripheral nerve maximum length (mm)	NS	NS	NS	NS	NS	NS	NS	NS	NS
	Corneal peripheral nerve average density (mm/mm^2^)	NS	NS	NS	NS	NS	NS	NS	NS	NS
	Average LC number of the corneal vortex (number)	NS	NS	NS	NS	NS	NS	NS	NS	NS
	Average LC number of the corneal periphery (number)	NS	NS	0.42*	NS	NS	NS	NS	NS	NS
	Average LC number of total cornea (number)	0.41*	NS	0.54**	NS	NS	NS	NS	NS	NS

*LC, Langerhans cell; The correlation coefficients (CC) are shown for all significant correlations (P < 0.05).*

*Significant correlation (P < 0.05); **Significant correlation (P < 0.01); NS, no correlation was detected during correlation analysis.

### Correlation Analysis Among Subbasal Nerve Parameters

The maximum length and average density of the corneal vortical nerve and the average density of the corneal peripheral nerve were observed to be strongly associated with peripheral LC number (for detailed information, refer to Table 5).

**TABLE 5:** Correlation analysis among sub-basal nerve parameters.

	Corneal vortical nerve maximum length (mm)	Corneal vortical nerve average density (mm/mm^2^)	Corneal peripheral nerve maximum length (mm)	Corneal peripheral nerve average density (mm/mm^2^)
Average LC number of the corneal center (number)	NS	NS	NS	NS
Average LC number of the corneal periphery (number)	−0.20**	−0.16*	−0.15*	NS
Average LC number of total cornea (number)	−0.16*	NS	NS	NS

*LC, Langerhans cell; The correlation coefficients (CC) are shown for all significant correlations (P < 0.05).*

*Significant correlation (P < 0.05); **Significant correlation (P < 0.01); NS, no correlation was detected during correlation analysis.

## Discussion

Through the use of IVCM, more knowledge about the microenvironment of the cornea, nerves, and cells under physiological and pathological conditions has been gained (23). However, there is a lack of immunohistochemical evidence for cell-type identification in confocal microscopy. It is well known that the cornea is an immune-privileged site; however, the privileged status is relative with regard to susceptibility to immune-mediated inflammatory diseases, such as ocular infections and autoimmune diseases (24). Macrophages in the stroma and dendritic cells (DCs) are involved in this process and serve as antigen-presenting cells (APCs) (25). They can engage in processing and presenting of antigens, resulting in the initiation of ocular surface immune-inflammatory responses. LCs are a unique subset of DCs found at a depth of 35–60 μm in normal human central corneal epithelium and act as first-line sentinels and professional APCs on the ocular surface. They are thought to be the only cells that constitutively express Iα molecules in the cornea. LCs are rarely found in the corneal center or present as immature phenotypes that lack dendrites under non-pathological conditions. However, those located in the cornea’s periphery show long interdigitating processes in the corneal epithelium (25). With the advancement of maturity, LCs undergo functional and morphological changes (26). To date, few studies have investigated the location of LCs in the corneal center. Recently, we observed bright, oval cells at the vortex, which differ in morphology from slender immature LCs and these cannot be macrophages as the latter are almost exclusively restricted to the posterior stroma (25). Hence, it was hypothesized that the oval cells are a particular type of LC that may be related to the severity of inflammation. *In vitro* experimental studies suggested that the corneal center comprises almost only immature LCs, whereas the corneal periphery contains both the mature and immature LCs (25). The normal uninflamed cornea contains many major histocompatibility complex class II-positive LCs that are in an immature state. When exposed to proinflammatory cytokines, cells harvest, lose their capacity to process antigens, and gain the ability to stimulate T cells in this process. Previous studies have suggested that harmful stimulation, such as cauterization or corneal transplantation, is associated with the maturation of resident corneal LCs (27). Morphological differences in corneal LCs are related to variations in their functional state (28). In this study, these oval cells were found in the corneal vortex of patients with DED. To demonstrate that oval cells are associated with DED severity, 168 patients with different degrees of DED were enrolled. Cases were grouped into three based on presence or absence of LCs: Group 1 with no LCs; group 2 had both the LCs and bright, oval cells; and group 3 had LCs, but no bright, oval cells. The IVCM approach seemed less invasive; however, it is effective as impression cytology (29).

Analysis of the three groups showed no significant differences in terms of age, sex distribution, DED symptoms, and signs. The evaluation of symptoms (possibly associated with inflammation), tears secretion and stability (possible reasons of inflammation), and superficial epithelial alterations (possibly brought on by inflammation) seem to provide complex information more suitable for disease classification rather than inflammatory activity assessment (29). Individual symptoms and signs cannot accurately reflect the severity of disease that has given us a better understanding of the inconsistent symptoms and signs of DED. In this study, there were no differences in symptoms and signs among the groups. In contrast, marked differences were found in the corneal peripheral nerve maximum length, average density, and average LC numbers. Previous studies have shown that the corneal subbasal nerve plexus density and length tended to decrease and tortuosity increased in patients with DED, indicating damaged nerve fibers (30). These results suggest that the changes in corneal nerve density and nerve number are related to the severity of dry eye condition. It was observed that group 2, which had both the LCs and bright, oval cells, had a greater corneal peripheral nerve maximum length and average density than the other groups. It can, thus, be inferred that increased nerve length and number occur under mild or moderate dry eye conditions. The results from this study are consistent with previous reports suggesting the regeneration of corneal nerves in patients with dry eye (31). In addition to increasing nerve number and density, nerve tortuosity increased and nerve sprouting was observed in dry eye, suggesting corneal nerve outgrowth (32). Nerve regeneration in DED may be attributed to ocular inflammatory responses. Long-lasting inflammation of the ocular surface stimulates the release of prostaglandins, leukotrienes, and cytokines, such as interleukin-1 (IL-1) and IL-6, from damaged corneal tissue and inflammatory cells (33–35). This leads to the activation of keratocytes, followed by the synthesis of nerve growth factor or other neuronal growth factors, such as ciliary neurotrophic factor (36). IL-6 can also contribute to neurotrophy (37). As a result, early in the clinical course of DED, corneal nerve regeneration increases the corneal length and density.

Meanwhile, we noticed that group 3, which had LCs, but no bright, oval cells, had less corneal peripheral nerve maximum length and average density than others, thus proving damaged corneal nerve fibers in DED. It is likely that the tear film was thinned and the mechanical stress generated by blinking became abnormally high, injuring the terminal nerve branches, thus contributing to nerve damage (38). Another possible reason for corneal nerve damage is continued exposure to inflammatory agents (39). Inflammation is a major driving force in sensitization, damage, and regeneration of peripheral sensory neurons (39). The appearance of nerve damage indicated that the disease was severe enough that the cornea could not repair it. The maximum length, average density of the corneal vortical nerve, and average density of the corneal peripheral nerve were inversely related to the peripheral LC number, which validated that inflammation plays a vital role in the pathogenesis and chronicity of DED.

Taken together, this may explain why previous studies reported controversial results of quantitative nerve changes in DED, from a decreased nerve number to no change in the nerve number or an increased nerve number. This is because of the different periods of the disease. The bright, oval cells may present that patients are early or middle in the process of developing DED.

**FIGURE 1:**
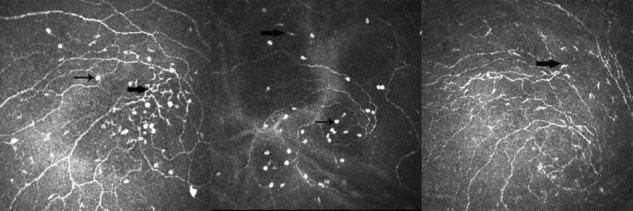
Example of different types of Langerhans cell (LC) in the corneal vortex of dry eye disease (DED). Thin arrow shows a type of bright and oval cell; thick arrow shows the differentiated LCs.

Recent studies showed a significant increase in the density of DC in some cases, such as infectious keratitis, DED, and contact lens wear (15, 40). Apart from density, the increase in size is accompanied by an altered morphology, which is the dendritic process lengthening (41). In this study, we determined whether the average LC number of the corneal vortex, corneal periphery, or total cornea was in the order of group 3 > group 2 > group 1. DCs were activated in both the animal models and patients with DED and corneal DC density increased (42). Disease severity can be inferred from the number of inflammatory cells. It was found that peripheral LC number was associated with symptoms and signs of DED, especially blurred vision and TMH in group 1, whereas in group 3, it was only associated with foreign body sensation. However, in group 2, vortical LC number was associated with TBUT. The inconsistency of these correlations showed that patients in each group had different disease severities and it was an excellent example of why some patients with dry eye have inconsistent symptoms and signs. In group 2, both the LCs and bright, oval cells had a moderate number of inflammatory cells; thus, we suspected that patients with bright, oval cells might be in the early or middle stage of DED. They are not only an immature type of DC but also an indicator of disease severity. Additionally, we found that these oval cells and LCs with dendritic processes are simultaneously present in the corneal vortex (Figure 1). It was assumed that they were at different stages of the same cell. Interestingly, we found that the nerve maximum length, average density, and average LC number of the corneal peripheries were significantly greater than those of the corneal vortex, which does not conflict with previous studies. This is because the corneal vertex is not located at the center of the cornea. The number of peripheral LCs was greater than that of central LCs. We do not know where the LCs came from, although the traditional view is that LCs are derived from the corneal periphery and migrate to the center. LCs may be stored under the corneal vortex and tend to move from the vortical part to the peripheral part when the cornea is stimulated (43). There are three reasons for this finding: First, immature LCs can be observed in the corneal vortex and the appearance of oval cells is related to DED severity. Second, there are more LCs in the periphery than in the vortex. Finally, the area under the corneal vortex could not be seen clearly by ICVM, which suggests that there may be unique structures under the corneal vortex, such as the cell storage pool. LCs are contiguous to nerves of the basal epithelial plexus in histopathologic specimens (44). Further characterization of LCs using immunohistochemistry may better validate our assumption, which is infeasible *in vivo*. The corneal nerves and corneal cells degenerate a few hours after death. This means that we need to obtain isolated fresh human cornea from normal individuals and patients.

### Limitations

Inevitably, this study has some limitations. First, patient follow-up to record the changes in oval cells before and after anti-inflammatory treatment was not carried out. Second, freshly isolated human corneas were difficult to obtain; however, animal experiments may provide clues of oval cells.

## Conclusion

A bright and oval cell type, which might be an immature LC, was identified in the corneal vortex. Its appearance is related to the severity of DED. This suggests that the LC may originate from the deep layer of the corneal vortex. Further study should focus on the corneal vortex, which is a unique structure.

## Data Availability Statement

The raw data supporting the conclusions of this article will be made available by the authors, without undue reservation.

## Ethics Statement

This study was approved by the Ethics Committee of the Peking University Third Hospital and was performed in accordance with the principles of the Declaration of Helsinki (#M2019236). The patients/participants provided their written informed consent to participate in this study.

## Author Contributions

DJ contributed to research design, data acquisition, data analysis, and manuscript preparation. XJ contributed to research design and manuscript modification. YC contributed to data acquisition and data analysis. SW and JS contributed to data acquisition. RH contributed to data analysis. XL contributed to research design. All authors contributed to the article and approved the submitted version.

## Conflict of Interest

The authors declare that the research was conducted in the absence of any commercial or financial relationships that could be construed as a potential conflict of interest.

## Publisher’s Note

All claims expressed in this article are solely those of the authors and do not necessarily represent those of their affiliated organizations, or those of the publisher, the editors and the reviewers. Any product that may be evaluated in this article, or claim that may be made by its manufacturer, is not guaranteed or endorsed by the publisher.
